# Metabolomics in Plant Priming Research: The Way Forward?

**DOI:** 10.3390/ijms19061759

**Published:** 2018-06-13

**Authors:** Fidele Tugizimana, Msizi I. Mhlongo, Lizelle A. Piater, Ian A. Dubery

**Affiliations:** Department of Biochemistry, Research Centre for Plant Metabolomics, University of Johannesburg, Auckland Park 2006, South Africa; Fideletu@gmail.com (F.T.); msizi.mhlongo17@gmail.com (M.I.M.); lpiater@uj.ac.za (L.A.P.)

**Keywords:** metabolomics, plant defence, plant–microbe interactions, priming, pre-conditioning

## Abstract

A new era of plant biochemistry at the systems level is emerging, providing detailed descriptions of biochemical phenomena at the cellular and organismal level. This new era is marked by the advent of metabolomics—the qualitative and quantitative investigation of the entire metabolome (in a dynamic equilibrium) of a biological system. This field has developed as an indispensable methodological approach to study cellular biochemistry at a global level. For protection and survival in a constantly-changing environment, plants rely on a complex and multi-layered innate immune system. This involves surveillance of ‘self’ and ‘non-self,’ molecule-based systemic signalling and metabolic adaptations involving primary and secondary metabolites as well as epigenetic modulation mechanisms. Establishment of a pre-conditioned or primed state can sensitise or enhance aspects of innate immunity for faster and stronger responses. Comprehensive elucidation of the molecular and biochemical processes associated with the phenotypic defence state is vital for a better understanding of the molecular mechanisms that define the metabolism of plant–pathogen interactions. Such insights are essential for translational research and applications. Thus, this review highlights the prospects of metabolomics and addresses current challenges that hinder the realisation of the full potential of the field. Such limitations include partial coverage of the metabolome and maximising the value of metabolomics data (extraction of information and interpretation). Furthermore, the review points out key features that characterise both the plant innate immune system and enhancement of the latter, thus underlining insights from metabolomic studies in plant priming. Future perspectives in this inspiring area are included, with the aim of stimulating further studies leading to a better understanding of plant immunity at the metabolome level.

## 1. Introduction: Multi-Layered Molecular and Cellular Networks Ensure Effective Adaptation to Changing Environments

Evolution dictates that living systems constantly adapt to ever-changing environments in a context-dependent manner. Such adaptation and/or response to environmental or genetic alterations implies complex and dynamic cellular reprogramming [1,2,3]. These biological responses—which can be phenomenologically described by understanding the cellular or organismal physiological state—are kinetic and highly dynamic events that span the whole cellular biological information network [4,5,6,7]. Reflecting on the plant kingdom, one of the epitomes of such adaptation is the constant fine-tuning of physiologies and cellular-scale morphologies, and the dynamic (and complex) biosynthesis of an array of structurally and functionally diverse chemistries [8,9,10].

Plants are seemingly as adept as animals in responding to environmental conditions. Of necessity, considering their sessile nature, plants have developed dynamic, multi-layered molecular and cellular networks for effective adaptation to unpredictably changing environments [10,11,12]. The latter is a natural habitat of the continuously-evolving pathogenic microorganisms that represent a biological threat to food security [13,14,15]. Furthermore, the sustainable production of food plants, considering the exponentially growing world population, is currently one of the challenges facing humanity. Crop losses due to plant pathogens can be quite substantial, with far-reaching effects. Moreover, most of the classical methods for crop protection against pathogenic microorganisms have become less effective and are environmentally unfriendly. Hence, the need for new strategies has led to procedures/metabolites that aid plants in adapting and defending against stress [16,17].

Learning from nature, plant biologists have observed that the interaction of plants with necrotising pathogens, beneficial microbes or agrochemicals can cause a sensitisation of the plant immune system, resulting in a faster and stronger induction of resistance mechanisms upon subsequent infections [18,19,20,21]. Memory of a past event may determine the response to future environmental stimuli, thus resulting in phenotypic and stimulus-dependent plasticity of response traits [22]. This unique physiological state in which plants are rendered capable (pre-conditioned) to better or more effectively mount defence responses to biotic or abiotic stresses is termed ‘priming’ [20,23,24], and differs from adaptation and acclimatisation phenomena in response to environmental stimuli [22]. In view of the competition of plant resource allocation to defence vs. growth, priming demands a small fitness cost that is, however, considerably surpassed by the benefits of an enhanced defensive capability to ward off attacks by potential pathogens [22]. Furthermore, a primed defence state can be inherited epigenetically from defence-expressing plants [19,25,26,27,28].

This immune stimulation of plants could be an alternative strategy that holds promise for increasing the capacity of plants to cope with biotic and abiotic stresses. Most of the efforts in characterising and defining the biochemical changes related to priming processes have been driven by targeted approaches. Although these methodologies have played a vital role in elucidating the main elements of priming that includes enhanced perception systems, dormant signal transduction enzymes, chromatin modification and transcription factors [19,23,29,30,31], there are still gaps in holistically understanding the dynamism and complexity of molecular mechanisms involved in the entire priming event, considering the complexity of multi-layered biological information networks. The phenomenological description of the physiological responses defining the ‘prime-ome’ is thus rendered possible by systems biology approaches (-omics layers—genomics, transcriptomics, proteomics and metabolomics) [22,24,32,33].

## 2. Metabolomics, a Systems Biology Approach: Prospects and Challenges

A recent resurgence of interest in metabolism and increasing awareness about the physiological insights that can be obtained by measuring the total small-molecule complement of a biological system have made metabolomics a central pillar in systems biology approaches [2,34,35]. Metabolomics can thus be understood as a quantitative measurement of the multi-parametric metabolic responses of living systems to genetic or environmental perturbations [36,37,38]. Such a description implies that metabolomics can be regarded as the best trade-off for ‘functionally’ investigating metabolism, offering the finest-grained details: a molecular-level convolution of all upstream biological information (genomic, transcriptomic and proteomic) layers [38,39,40,41].

**Figure 1:**
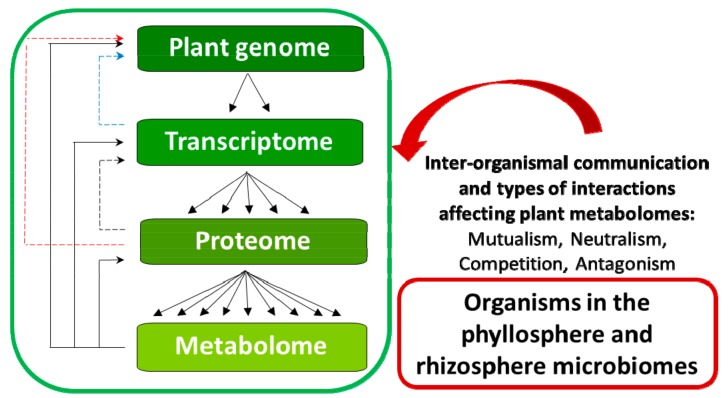
Metabolomics in the context of biological information flow, illustrating the complexity of multi-layered biological information networks and mutual interdependence. In biological systems, large numbers of structurally and functionally diverse genes, proteins and metabolites are involved in dynamic, linear and/or non-linear interactions. These interactions may involve a range of time scales and intensities. Some of the types of reciprocal interactions include post-transcriptional control of gene expression (dotted lines). Others include effects of downstream metabolites on transcription through binding to regulatory proteins and feedback inhibition/activation of enzymes (solid lines). Adaptive gene expression in response to environmental influences is ultimately reflected in changes in the pattern and/or concentration of metabolites.

These small molecules (namely metabolites, with molecular masses ≤1500 Da) can be described as the end products of gene expression and define the phenotype of a cell or tissue under defined physiological conditions at a biochemical level. Metabolite profile patterns can thus provide a holistic signature of the physiological state under study as well as deeper knowledge of specific biochemical processes [42,43,44,45]. Furthermore, systems biology approaches imply an appreciation of the full complexity and the multi-dimensionality of biochemical networks operating in a biological system to produce physiological and phenotypic coherence (Figure 1). Hence, given that the biochemical actions of metabolites are far-reaching, including regulation of epigenetic mechanisms and gene expression, involvement in signal transduction, post-translational modifications of proteins, protein transport and active roles in defence mechanisms; metabolomics can thus be seen as a powerful tool to investigate cellular biochemistry at the systems level [46,47,48,49].

In this review, the term “metabolomics” refers to an untargeted methodology [34,45]. Thus, metabolomics and untargeted or non-targeted metabolomics may be used interchangeably to mean the global metabolic profiling of the entire (measurable) metabolome of the biological system under consideration. This methodology differs from targeted analytical methods in various fundamental aspects such as being a data-driven approach with predictive power that aims to assess (qualitatively and quantitatively) all measurable metabolites without any pre-conception or pre-selection [41,45,50,51].

Being at the interface between biology, chemistry, chemometrics, statistics and computer science; metabolomics is methodologically a multi-disciplinary skillset research field [40,45]. With the innovative developments in analytical technologies, advancement in chemometric and statistical methods, and the integration of orthogonal biological approaches, metabolomic studies have provided remarkable insights into the biochemical mechanisms that underpin various physiological conditions [51,52,53]. Furthermore, owing to the inherent sensitivity of the metabolome to genetic and environmental perturbations, subtle alterations in biological pathways can be measured [41,50,54,55].

**Figure 2:**
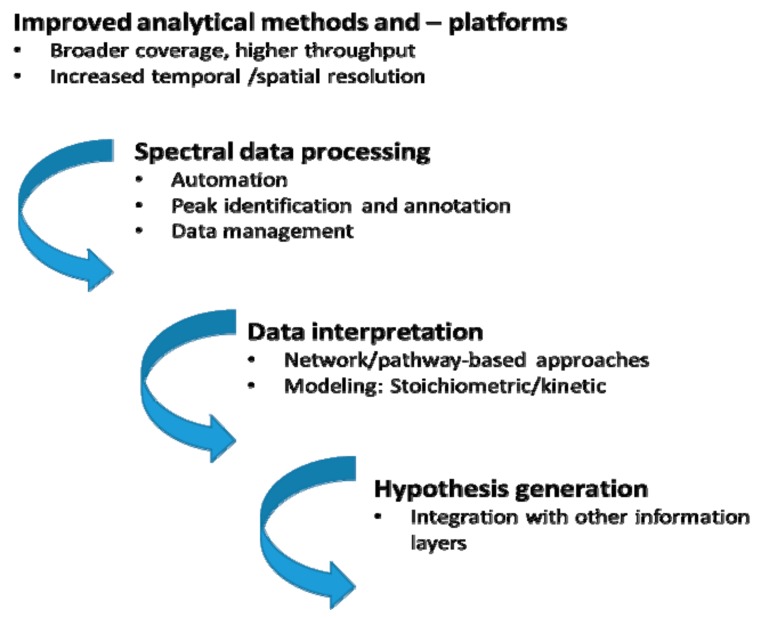
Bottlenecks in metabolomics workflows that limit biological insights. Despite the maturation of metabolomics, driven by massive improvements in analytical technologies and impressive advancements in computational and chemometric methods, the realisation of the goal of metabolomics is still a challenge at different levels: metabolome coverage; information extraction from acquired data; and systematic interpretation of complex metabolic changes and derived hypotheses about underlying functional mechanisms.

To attain this goal—holistic analysis of the metabolome, considering the complexity and chemo-diversity thereof—a wide range of chemistries, chemometrics methods, novel computational approaches and advanced analytical instrumentation that provide high degrees of sensitivity and reproducibility, are required and employed in metabolomics [56,57,58,59]. In contrast to other -omics methodologies, metabolomics faces several unique challenges that make the field particularly demanding. These arise from the inherent characteristics of the metabolome: highly dynamic (continuously changing at different rates), chemically diverse (dramatically different physicochemical properties and biological functions of metabolites, as well as highly diverse and dynamic stereochemistries), a wide range of metabolite levels and the inherent bio-complexity of living systems (biological cycles, organismal and cellular compartmentalisation) [40,45,60,61]. These challenges point to the bottlenecks that have limited (untargeted) metabolomics so far (Figure 2), making the holistic coverage of the whole metabolome currently unrealisable and subsequently impacting the biological insights generated.

These holdups can be summarised into three main aspects related to different steps of the metabolomics workflow pipeline. Firstly, the analytical limitations, be it at the metabolite extraction [62,63,64] or analytical platform level [65,66,67,68], hinder the metabolome coverage. The two leading and successful analytical platforms in metabolomics are mass spectrometry (MS) and nuclear magnetic resonance (NMR) spectroscopy. MS platforms provide high sensitivity and detection specificity, thus enabling large-scale coverage of the metabolome. NMR, on the other hand, offers a window into profiling all the most abundant metabolites in sample extracts; and the strengths of this analytical platform include detection of poorly ionisable compounds, identification of compounds with identical masses, and determining structures of unknown metabolites [45,69]. Evidently, MS (often coupled to chromatographic separation) and NMR approaches offer different advantages, which are being explored synergistically [70,71]. However, despite the current analytical advancements, and considering the inherent complexity of metabolomes, the realisation of holistic coverage of the metabolome in toto, in a given biosystem, is not yet possible [69,72].

Secondly, extracting information from acquired data is still a major challenge, thus limiting the maximisation of the value of metabolomics data. This can be at the level of data processing steps: from pre-processing to chemometric and statistical analyses [34,56,58,73,74]; and at the systematic identification of metabolites, with accuracy and high confidence levels [72,75,76,77]. Thirdly, data interpretation and hypothesis generation are still a bottleneck, as comprehensive strategies (computational and chemometrics) are still limited, and the integration of other biological information layers is still in the developmental phase and has limitations [3,54,78,79,80]. A detailed description of these three constraints can be found in the cited literature herein.

Despite these challenges, the momentum and maturation of metabolomics have visibly revolutionised life sciences. Innovative and collaborative efforts are continuously providing suggestions to address these limitations: technological advancement, data mining strategies and tools, systematic data interpretation and integration of orthogonal biological information [72,73,76,81,82].

The application of metabolomics spans a wide spectrum of life sciences (fundamental and translational) research [40,44,45]. In the plant sciences, metabolomic approaches are increasingly being used for investigating linkages between genotype and biochemical phenotype [42,83,84], metabolic pathway studies [85,86,87], silent phenotypes of mutations [6,88], plant–pathogen interactions [89,90,91,92,93] and, as emphasised here, plant priming [24,49,94]. As indicated in the above sections, an overview and reflections on plant–pathogen interactions and priming are articulated, highlighting the metabolomics inputs in decoding the plant priming molecular events. Thus, the following section gives a succinct overview of plant defence mechanisms, briefly highlighting key components and current models that describe plant defence responses.

## 3. Plant Defence Mechanisms—Core Concepts, Key Molecular Components and Current Models

Due to co-evolution between plant hosts and pathogens, plants have developed sophisticated abilities to recognise pathogens and translate this perception into effective immune response strategies for survival. Concurrently, pathogens have evolved their own strategies to evade the host’s immune system. The plant–pathogen interactions are undeniably a never-ending evolutionary arms race, and involve key elements for the survival of the host or the pathogen [95,96,97,98]. Understanding these interactions at the biochemical level is necessary to develop strategies to aid plants to adapt and defend against continuously evolving pathogens. It may suffice here to briefly point out some classical and current components that define the plant defence mechanisms.

**Figure 3:**
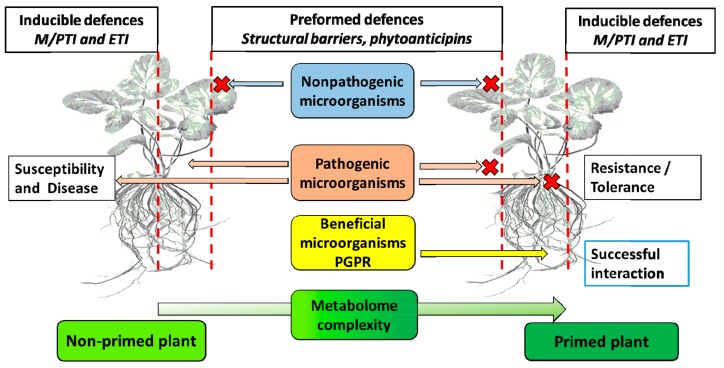
Priming, plant–microbe interactions and innate immunity. Physical barriers (waxes, suberin, callose, lignin) and innate immunity defences (MTI, MAMP-triggered immunity, and ETI, effector-triggered immunity, indicated by vertical red lines) may affect priming by biotic inducers. Interactions can either be disease-related due to biotrophic or necrotrophic pathogenic microorganisms, or beneficial due to plant growth-promoting rhizo microorganisms (PGPR) interactions with plant roots. The red crosses indicate the inability of the interacting microbe to overcome the line of defence.

Plant cells are generally protected by an array of structural barriers (waxes, suberin, lignin, etc.) that deny access to a wide range of microbes (Figure 3). This passive protective system can also involve preformed antimicrobial chemicals (metabolites known as phytoanticipins) that form a chemical barrier, in so doing preventing or attenuating invasion by potential attackers [99,100,101,102]. In addition to these non-specific defence mechanisms, active immune responses can be activated by the perception of highly conserved molecular features of different classes of bacterial and fungal pathogens, referred to as microbe/pathogen-associated molecular patterns (M/PAMPs) [103,104]. These non-self M/PAMPs are recognised by cell surface-localised pattern recognition receptors (PRRs), which are activated and, in turn, initiate downstream signalling events that ultimately result in the activation of a defence response referred to as M/PAMP-triggered immunity (M/PTI) [105,106,107]. These responses include newly synthesised antimicrobial metabolites (phytoalexins), antimicrobial hydrolytic enzymes (e.g., ß-glucanases, chitinases) and small-molecule precursors of cell wall-strengthening polymers. In this evolutionary arms race pathogens, on the other hand, can suppress M/PTI by transporting effector molecules into the host cell to target response regulatory components of the immune system. Furthermore, the invading pathogens can be protected by surface polysaccharides, and can produce antioxidants and enzymes to scavenge or detoxify the M/PTI-related toxic reactive oxygen species (ROS) [108,109,110,111,112].

To counter this infection strategy, plants have evolved specialised immune receptors encoded by resistance (*R*) genes that recognise these pathogen-specific effectors, thereby leading to an amplified secondary immune response known as effector-triggered immunity (ETI) [113,114,115]. ETI is mostly characterised by the induction of localised programmed cell death (referred to as the hypersensitive response or HR) in order to limit the spread of the infection, activation of defence gene expression, induction of local induced/acquired resistance (LAR) to contain the invader at the infection site, and systemic acquired resistance (SAR), which induces defences in distal, non-infected parts of the plant [116,117,118]. M/PTI, as the first facet of active plant defence, is the primary driving force of plant–pathogen interactions, and has been shown to confer resistance to a wide spectrum of pathogens [102,103,119]. Furthermore, experimental evidences indicate that, in some instances, input and output responses of both M/PTI and ETI converge, pointing to an interplay between M/PTI and ETI to coordinate plant immunity [120,121,122].

The active plant resistance system is highly complex and involves the coordination of a myriad of highly regulated mechanisms, with phytohormone crosstalk networks as central signalling systems [46,123,124,125]. These cellular (and biochemical) processes are spatially organised and highly controlled in intracellular compartments, and temporally complex due to the highly dynamic nature [126,127,128]. The molecular mechanisms of these prompted immune responses are not yet fully understood. Current models indicate that plant defence mechanisms involve cellular and organismal reprogramming expressed at the interconnected systems layers that collectively define the defensive metabolism (the ‘defensome’) and subsequent physiological state [24,129,130,131]. Thus, the ability of the pathogen to suppress the immune system of the host, and the capacity of the plant to recognise the pathogen and activate effective defences, define the outcome of the plant defence reaction.

Although plant defence immunity has been extensively studied and some aspects have been thoroughly explored, providing a wealth of detailed biochemical insights that have shaped our understanding [33,103,132,133,134], the plant–pathogen interactions field still has grey areas and remains an active field of research. Thus, elucidating host receptors and regulatory mechanisms that determine certain responses, unravelling biochemical processes in specific phytopathosystems, elucidating the environmental influences on diverse phyllosphere and rhizosphere interactions with microorganisms, and illuminating epigenetic regulatory mechanisms that result in passing on defensive traits to the progeny are some of the topics that still need to be fully explored.

## 4. Plant Priming: What Drives the Pre-Conditioned State

Lacking specialised mobile immune cells, every plant (and cell) is theoretically capable of establishing an active immune response upon an attack. Of necessity, such a plant immune system is characterised by self-surveillance, systemic signalling and genetic changes as mechanisms to provide successful protection and transgenerational survival [28,135]. Thus, one mechanism by which plants can enhance their resistance capacity is by potentiating the responsiveness of the immune system upon recognition of some danger-related signals from the environment. This phenomenon is known as “priming” or “pre-conditioning”, and can be described as an induced state whereby a plant is pre-exposed to an inducing agent, thus rendering it more resistant to secondary stresses, i.e., the “primed” plant responds more rapidly and/or more efficiently to a subsequent stress [23,24,30,33]. Priming agents thus act as response modifiers that can lead to a more intense defence response, a faster response, an earlier response or a more sensitive response compared to the non-primed response to the same stress condition [22].

**Figure 4:**
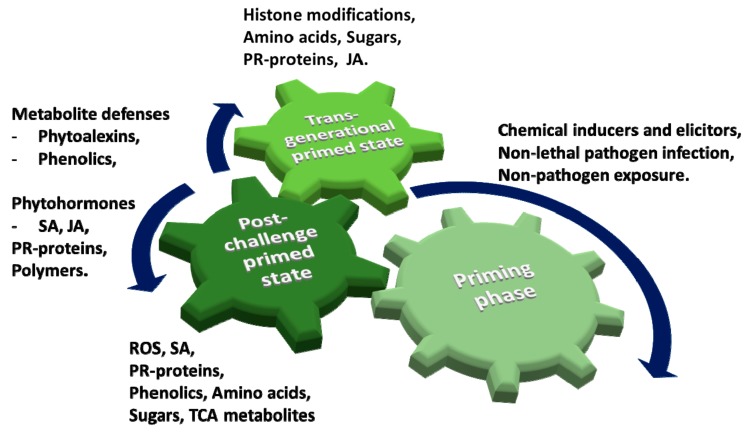
Phases in priming events. Priming generally requires sequential environmental stimuli. The priming phase is initiated by a triggering stimulus to last until the plant is exposed to a challenging stress. During this phase, slight alterations in the levels of primary—and secondary metabolites, (e.g., phytohormones, SA and JA) place the plant in a standby state of alertness. When challenged with a secondary stress, primed plants move on to the post-challenge primed state, associated with the induction and rapid deployment of defence reactions. This involves de novo biosynthesis of antimicrobial compounds. Primed plant can revert to the naïve state, but a transgenerational primed state may occur in plants when inherited from primed parental plants [12,22,24,33].

Priming can occur as a result of interactions between the host plants and beneficial microorganisms (rhizobacteria, mycorrhizal fungi) or virulent/avirulent pathogens, or by natural or synthetic compounds such as certain agrochemicals. Following such interactions, plants are cellularly and organismally reprogrammed in a long-lasting manner, and “remember” such events at a molecular level. Depending on the initial stimulus and the target of priming, primed plants can deploy a diverse set of defence mechanisms that are more rapid and stronger compared to non-primed plants [24,136,137,138,139]. Spatially, the priming events involve multiple cellular compartments, and this induced state temporally consists of three stages (Figure 4) namely (i) the priming phase (perception of stimulus), (ii) the post-challenge primed state (challenges by secondary stimulus) and (iii) the transgenerational primed state (primed state inherited from primed parents) [19,24]. These different physiological states (naïve, primed and primed and pathogen-triggered) are reflected in changes to the metabolomes and can best be investigated through untargeted metabolomics approaches [45].

Priming can involve various layers of induced defence mechanisms that are active during different levels of plant–pathogen interactions. Such mechanisms result in a broad span of effectiveness: ranging from early responses controlled by changes in hormone-dependent signalling pathways to longer lasting mechanisms involving chromatin modification and DNA methylation. Despite the grey areas in the mechanistic understanding of priming events; some characteristic key features of priming processes have been elucidated and include ‘memory’, low fitness costs and more robust defences. These are associated with enhanced levels of PRRs, proteomic and metabolic reprogramming and histone modifications and resulting chromatin changes [22,28,33]. As highlighted in Figure 4, studies have indicated that the priming status of a plant can be inherently stored and passed on to its offspring, lasting for few generations and conferring improved defence responses and resistance to biotic and abiotic stresses [140,141,142]. This transgenerational primed state implies mechanisms ranging from epigenetic marks to the accumulation of (dormant) defence-related molecules [22,140,141,142].

Furthermore, as an induced state of resistance in plants, priming can be a result of SAR or induced systemic resistance (ISR) or other forms of induced resistance mechanisms [30,33,143]. In simplified terms, SAR is currently understood as the salicylic acid (SA)-dependent process, involving the transduction protein NPR1 to establish a defensive state in uninfected systemic plant parts [116]. On the other hand, ISR is induced by beneficial microbes (such as growth-promoting rhizobacteria and fungi) and orchestrates a defensive state that depends on other hormones such as jasmonate (JA) and ethylene (ET). However, studies have demonstrated that the induced resistance (IR) state involves interconnected mechanisms (more than just SAR and ISR), creating a network of defences that define the plant immune system in toto. The biochemical and molecular details of these mechanisms are still not yet fully formulated; however, with the advent and progress in systems biology methodologies, in-depth insights are gradually being achieved [138,144,145,146].

These IR mechanisms imply reprogramming of cellular metabolism and the reciprocal crosstalk with cellular regulatory machinery. Profiling metabolic changes is providing a wealth of descriptive information that advances our understanding of priming mechanisms. These emerging endeavours have pointed to the reprogramming of primary metabolism and differential biosynthesis of secondary metabolites as characteristic processes involved in priming events [12,20,26,49]. Experimental evidences have shown that priming of *Arabidopsis thaliana* plants by β-aminobutyric acid (BABA) involves alterations in tricarboxylic acids fluxes involving malate, oxoglutarate and fumarate, and the intensification of phenylpropanoid biosynthesis and the octadecanoic pathway [146].

Comparative metabolomic analyses of chemically-induced priming by BABA in *Arabidopsis* plants also demonstrated that the resultant defence priming significantly affected sugar metabolism, cell-wall remodelling and levels of shikimic acid derivatives. This metabolic reprogramming resulted in specific changes in amino acid profiles and accumulation of camalexin, indole-acetic acid and indole-3-carboxaldehyde [147]. Similarly, an untargeted metabolomics analysis of *A. thaliana* treated with a pathogen-derived priming agent, bacterial lipopolysaccharides, detailed the importance of tryptophan-derived indolic metabolites that included camalexin, indoleglucosinolates, indole-3-carboxylic acid and indole-acetic acid [92]. Furthermore, treatment of cultured tobacco cells with different chemical—(acibenzolar-S-methyl, azelaic acid and riboflavin) and pathogen-derived (chitosan, lipopolysaccharides and flagellin) inducers resulted in differential metabolic changes involving early phenylpropanoid pathway intermediates and products. The activation hereof is shown to be an important aspect of priming events, as well as the alterations in secondary metabolism pathways involving conjugation of hydroxycinnamic acid derivatives to quinic acid, tyramine, polyamines or glucose [49]. On the other hand, metabolic analysis of resistant progeny has shown that transgenerational priming is associated with enhanced levels of primary metabolites such as amino acids and sugars [20,22,148,149]. However, the altered pathways are highly dependent on the pathogen characteristic, i.e., biotrophic stimuli seems to mainly impact primary metabolism and involves SA signalling, while insects and necrotrophic fungi trigger secondary metabolism via JA/ET-dependent pathways [150]. A recent study has demonstrated that the symbiotic relationship between *Microbacterium* sp. 3J1 and pepper plants confers to the latter protection against drought through a metabolic reprogramming that involves production of osmo-protectants and antioxidants. These metabolic alterations spanned changes in C and N metabolism, resulting in increased levels of sugars and amino acids, phenolics and lignin precursors [151]. Another recent work, involving the interaction of poplar roots with the ectomycorrhizal fungus *Laccaria bicolor*, revealed the systemic adjustment of defence mechanisms in leaves, comprising transcriptional and metabolic reprogramming: enhancement of chitinases, volatiles, nitrogen-bearing compounds and decreased levels of phenolics. This mycorrhiza-primed state influences aboveground plant–insect interactions, conferring protection to the plant [152]. Table 1 gives a summary of available metabolic information on the priming phase, challenged phase and trans-generational priming induced by different stimuli.

These metabolomic studies (and other cited literature) indicate that multiple metabolic pathways are involved in the priming phenomenon. The interconnectedness of metabolic pathways that initially might seem distinct will increasingly be shown to have feedback loops that allow for quick activation of cellular defences to potential attackers present in the external environment, attempted ingress and resultant cellular damage. Furthermore, the combination of a multitude of biotic and abiotic stresses that plants face, and adaptability of priming events, have made the elucidation of the underlying molecular mechanisms a challenging endeavour. Thus, despite overlaps and similarities, priming mechanisms can vary and the same phenotypic traits might be the result of unrelated underlying events [24,138,139,140,141,142,143,144,145,146,147,148,149,150,151,152]. Unfortunately, most of the reported studies often assess just a few defensive traits related to priming events and overlook the overall multi-layered mechanisms of naïve versus primed plants.

Hence, elucidation of molecular mechanisms in priming events remains an active area of research. Knowledge regarding intracellular metabolic networks that define the dynamic metabolism of priming processes in a biosystem, pinpointing common and unique specific biochemical traits characterising the primed state across species, is crucial for translational applications from model plants to food and industrial crop plants.

## 5. Concluding Remarks and Perspectives

In this review, we give a brief overview of the current mechanistic understanding of the plant innate immune system related to priming. Furthermore, the contribution of metabolomics in plant priming studies is highlighted, pointing out where metabolomics can contribute to new insights and deeper knowledge. Through increased technological advances, scientists are now better equipped to study the detailed metabolomic changes associated with the underlying biochemical mechanisms that support priming. In addition, the inherent constraints of this -omics methodology that represent challenges to be addressed, are discussed.

As emphasised herein, defence priming is a complex natural phenomenon that pre-conditions plants for enhanced defence against a wide range of pathogens. As such, it represents a sustainable alternative or complementary strategy that can provide avenues for plant protection against disease. However, a comprehensive functional and mechanistic understanding of the various layers of priming events is still limited and hence offers opportunities for future research. Even though such studies are still few in number, metabolic profiling of primed and naïve plants interacting with pathogens have certainly provided highly informative insights.

The metabolomic studies thus far performed on priming-related scenarios indicate that this strategy might involve multiple pathways and that the induced resistance state is often broadly specific, and may vary from species to species and in different stressor–plant systems, thereby leading to different outcomes. Depending on the initial stimulus and the target of priming, primed plants can deploy a diverse set of defence mechanisms. This adaptability—‘stimulus-dependent plasticity of response traits’ [22]—of priming events makes it difficult to exactly define underlying mechanisms. Conversely, despite possible overlaps or similarities, priming mechanisms can and do differ, and the same phenotypic traits might be the result of unrelated causal events. It is thus apparent that priming can involve various ‘layers’ of induced defence mechanisms that are active during different ‘levels’ of plant–pathogen interactions.

Major questions regarding priming remain, and here metabolomics techniques and approaches can assist. These include (i) how the molecular dialogue between plant and priming agent (particularly plant-beneficial microbes) drives enhanced stress resistance, yet still benefits plant development; (ii) the switching from normal growth to defence activation and subsequent deactivation of the triggered defensive state; (iii) the dynamic traits of the defensive metabolism that describe the transportation of induced resistance signals (to distal parts of the plant and neighbouring plants) where the interactions between different metabolic networks in a spatial and temporal context need to be dissected; and (iv) transgenerational priming knowledge still needs to be exploited. Furthermore, environmental influences can affect how plant genetic programmes are realised and managed, thus controlling the metabolomic phenotype. Meta-metabolomics, targeted at the phytobiome should therefore be a future approach, i.e., inter-kingdom metabolomics aimed at unravelling the complexity of chemical communication in the rhizosphere or phyllosphere.

Metabolomics, as applied in the plant sciences, is progressing beyond biomarkers towards mechanisms. Here, chemometrics and network analysis can identify participating pathways, and stimulation of pathways can be detected by comparison of metabolite profiles with subsequent quantification of discriminatory biomarkers. Moreover, comparative studies of conserved and unique metabolic pathways from different phyla (phylametabolomics) will help in the annotation of metabolites as well as pointing to important new targets of investigation in plant priming studies.

A recent and future development is that of genome-scale models of metabolism to simulate and comprehensively analyse the metabolism of cells. To attain this, algorithms that use inputs from various-omics data types are used to construct cell-line and tissue-specific metabolic models from genome-scale models. However, it may be more challenging to accurately simulate metabolism in higher plants due to some enzymes being only active in specific cell or tissue types. In addition, it is still unclear how algorithm and parameter selection (e.g., gene expression thresholds, metabolic constraints) would affect model content and predictive accuracy. Further new developments are geared towards a framework for the de novo prediction of metabolic capabilities of a cell or tissue, based on its gene expression and metabolomic profiles. These insights will guide and promote development of tissue- and cell type-specific models, and enable researchers to predict a cell’s phenotype from the genotype.

## Figures and Tables

**Table 1:** An overview of different stimuli and examples of metabolic changes involved in plant priming.

Priming Agent	Plant	Phase	Classes of Induced Compounds	References
Beta aminobutyric acid (BABA)	*Arabidopsis thaliana*	Priming	TCA metabolites, amino acids, phytohormones, purines, cinnamic acid derivatives and fatty acids. Amino acids, indole compounds, polyamines, SA, ABA	[20]
		Secondary stimulus	-***Plectosphaerella cucumerina*** Enhanced levels of amino acids, indolic compounds and polyamines. SA downregulation, enhanced levels of JA and JA-Ile.	[147]
Hexanoic acid	*Solanum lycopersicum*	Priming	Fatty acids, oxylipins, phospholipids, chlorophyll metabolism (pheophorbide A), purines (adenosine 2′-monophosphate), sugars. Downregulation of TCA intermediate (citrate) and some amino acids.	[153]
		Secondary stimulus	-***Botrytis cinerea*** Glycolytic intermediated and sugars, fatty acids, ascorbate metabolism. Proline downregulation.	[153]
		Secondary stimulus	-***Pseudomonas syringae*** Serine upregulation. Downregulation of fatty acids, phytohormones (abscisic acid), signalling molecules (pipecolic acid) and amino acids (valine and threonine).	[153]
Lipopolysaccharide (LPS)	*Arabidopsis thaliana* leaves and cells	Priming	Phytohormones (SA and JA) and their methyl esters and sugar conjugates, glucosinolates, indolic compounds, cinnamic acids derivative and other phenylpropanoids.	[92]
LPS, chitosan and flagellin flg22	*Nicotiana tabacum* cells	Priming	Hydroxycinnamic acid conjugates of quinic acid, shikimic acid, tyramine, polyamines or glucose.	[49]
Acibenzolar-S-methyl, azelaic acid, riboflavin	*Nicotiana tabacum* cells	Priming	Cinnamic acid derivatives conjugated through ester and amide bonds.	[49]
Phenylacetic acid produced by *Bacillus fortis* IAGS162	Tomato	Priming	Amino acids and sugars.	[154]
		Secondary stimulus	-***Fusarium* wilt** SA, sugars, amino acids, hexanoic acid, cinnamic acids (caffeic acid), shikimic acid, quinic acid, TCA metabolites, amino chlorocoumarin and methylquercetin.	[154]
*Pseudomonas fluorescens* SS101	*Arabidopsis thaliana*	Priming	Indolic compounds and glucosinolates.	[155]
*Rhizophagus irregularis*	Tomato roots	Priming	Upregulation of cinnamic acid derivatives (ferulic acid, coniferyl alcohol and *p*-coumaroyl alcohol), lignin, yatein and oxylipins, (Z)-jasmone, tuberonic acid, tuberonic acid-12-β-glycoside, methyl-tuberonic acid-12-β-glycoside. Downregulation of phenolic amino acids, some cinnamic acids derivatives (*p*-courmaric acid and *p*-coumaraldehyde) and α-linolenic acid.	[156]
*Funneliformis mosseae*	Tomato roots	Priming	Upregulation of cinnamic acid derivatives (ferulic acid, coniferyl alcohol and *p*-coumaryl alcohol), lignin, yatein, oxylipins, (Z)-jasmone, methyljasmonic acid, jasmonoyl-isoluecine, 13-hydroperoxy-9,11,15-octadecatrienoic acid (HPOT), tuberonic acid, tuberonic acid-12-β-glyc, methyl-tuberonic acid-12-β-glyc. Downregulation of phenolic amino acids, some cinnamic acid derivatives (*p*-coumaric acid and *p*-coumaraldehyde), α-linolenic acid.	[156]
*Microbacterium* sp 3J1	Pepper	Priming	Glutamine and α-ketoglutarate, osmoprotectants, antioxidants, sugars, amino acids, phenolics, lignin precursors.	[151]
Tobacco mosaic virus (TMV)	*Nicotiana tabacum*	Trans-generational state	Sugars and amino acids	[149]
